# Luteolin targets the AGE-RAGE signaling to mitigate inflammation and ferroptosis in chronic atrophic gastritis

**DOI:** 10.18632/aging.205969

**Published:** 2024-06-24

**Authors:** Nailin Zhang, Pingping Chen, Xiaoyan Liang, Jianhui Sun, Qiquan Liu, Shengjiang Guan, Qiao Wang

**Affiliations:** 1 Clinical Research Base Office, Hebei Provincial Hospital of Chinese Medicine, Hebei, China; 2 Department of Pharmacology, Hebei University of Chinese Medicine, Hebei, China; 3 Eighth Clinical Medical College, Guangzhou University of Chinese Medicine, Guangzhou, China; 4 Hebei Key Laboratory of Turbidity Toxin Syndrome, Hebei University of Chinese Medicine, Hebei, China; 5 Department of Spleen and Stomach Diseases, Hebei Provincial Hospital of Chinese Medicine, Hebei, China; 6 Pharmaceutical Department, Hebei Provincial Hospital of Chinese Medicine, Hebei, China; 7 Pharmacological Analysis Teaching and Research Department, Hebei Medical University, Hebei, China; 8 Key Laboratory of Integrated Chinese and Western Medicine for Gastroenterology Research, Hebei Provincial Hospital of Chinese Medicine, Hebei, China

**Keywords:** luteolin, chronic atrophic gastritis, AGE-RAGE signaling pathway, ferroptosis

## Abstract

Chronic atrophic gastritis (CAG) is a chronic inflammatory disease and precancerous lesion in stomach cancer. Abnormal activation cellular ferroptosis further damages gastric tissue, which is susceptible to inflammation. Luteolin has powerful anti-inflammatory and regulatory potential for cellular ferroptosis. We aimed to clarify the involvement of luteolin in inflammation and ferroptosis during CAG. Luteolin targets were searched to identify intersecting genes in the chronic atrophic gastritis disease database. The AGE-RAGE pathway is a potential target of luteolin for the treatment of chronic atrophic gastritis and a binding site between luteolin and RAGE was predicted through a computer simulation of molecular docking. We established a CAG rat model using N-methyl-N-nitro-N-nitroguanidine. The therapeutic effect of luteolin on CAG was detected using western blotting, qPCR, hematoxylin and eosin staining, lipid oxidation (MDA), and Fe^2+^ assays. Luteolin inhibited the AGE-RAGE signaling pathway and reduced the inflammatory response in gastric tissues. Additionally, luteolin downregulated the concentration of (MDA) and Fe^2+^, and CAG downregulated the expression levels of ACSL4 and NOX1 and upregulated the expression levels of FIH1 and GPX4 ferroptosis-related proteins, thus inhibiting the ferroptosis of gastric tissue cells, which had a therapeutic effect on CAG.

## INTRODUCTION

Gastric cancer ranks fourth in cancer incidence and second in cancer-related deaths, posing a serious threat to physical and mental health [1]. Chronic atrophic gastritis (CAG) is a common digestive system disease recognized as a precancerous lesion in gastric cancer [2]. CAG is usually associated with *Helicobacter pylori* infection, autoimmune reactions, and other factors, and its clinical manifestations include dyspepsia, upper abdominal pain, fullness after meals, and other symptoms [3]. Treatment of chronic atrophic gastritis mainly focuses on improving symptoms and inhibiting inflammatory responses [4]. Recently, some new drugs, such as immunomodulators and antioxidants, have shown efficacy in clinical trials [5, 6]. However, owing to their therapeutic and long-term side effects, further development has limitations [7, 8]. Therefore, it is important to identify more specific drugs with low toxicity and high efficacy to reverse CAG symptoms and reduce the incidence of gastric cancer.

Natural drugs can be used for developing low-toxicity and high-efficiency targeted drugs [9, 10]. Luteolin is a naturally occurring flavonoid widely found in a variety of plants, such as comfrey and celery [11]. Recently, substantial progress has been made in the study of the pharmacological action of luteolin, which has a strong anti-inflammatory effect, inhibiting the expression of inflammatory factors, such as IL-1β and TNF-α [12]. Its anti-inflammatory mechanism may be related to the inhibition of signaling pathway activation, such as AGE-RAGE [13]. In addition, luteolin has good binding activity with differentially expressed genes in individuals with CAG [14]. Thus, luteolin is a potential drug for the treatment of CAG. However, the mechanism of action of luteolin in CAG treatment remains unclear.

Luteolin modulates iron cell death in different diseases, achieving different degrees of therapeutic effects [15]. Ferroptosis is a cellular stress response, recently attracting attention in many fields such as neuroscience, oncology, and immunology [16]. There is abnormal iron metabolism and ferroptosis in the gastric tissue of CAG [17]. Inducing gastric ferroptosis by inhibiting CAG can successfully hinder the progression of CAG [18], indicating that inhibiting CAG-induced ferroptosis is a new strategy for treating CAG. Luteolin inhibits ferroptosis in diseases, such as endometritis [19] and myocardial reperfusion injury [20]. However, whether luteolin activates or inhibits ferroptosis in CAG remains unclear. Therefore, our study focused on the initial mechanism of luteolin in the treatment of CAG and its relationship with ferroptosis, inflammation, and the AGE-RAGE signaling pathway in gastric tissue, with the aim of developing new natural drugs and providing new therapeutic strategies for the treatment of CAG.

## RESULTS

### Screening of potential therapeutic targets of luteolin in CAG

The GSE153224 and GSE191139 datasets were downloaded from the GEO database (https://www.ncbi.nlm.nih.gov/gds). We analyzed the principal components of the two datasets, as shown in Supplementary Figure 1A. Subsequently, we plotted a volcano map of differential genes (Supplementary Figure 1B) and found that 2,732 differential genes were upregulated and 2,359 differential genes were downregulated. We obtained the TOP20 genes with the most pronounced differences to plot a clustered heat map (Supplementary Figure 1C). We did not find information of interest using the gene ontology (GO) functional enrichment map (Supplementary Figure 1D) or Kyoto Encyclopedia of Genes and Genomes (KEGG) functional enrichment map (Supplementary Figure 1E).

**Figure 1:**
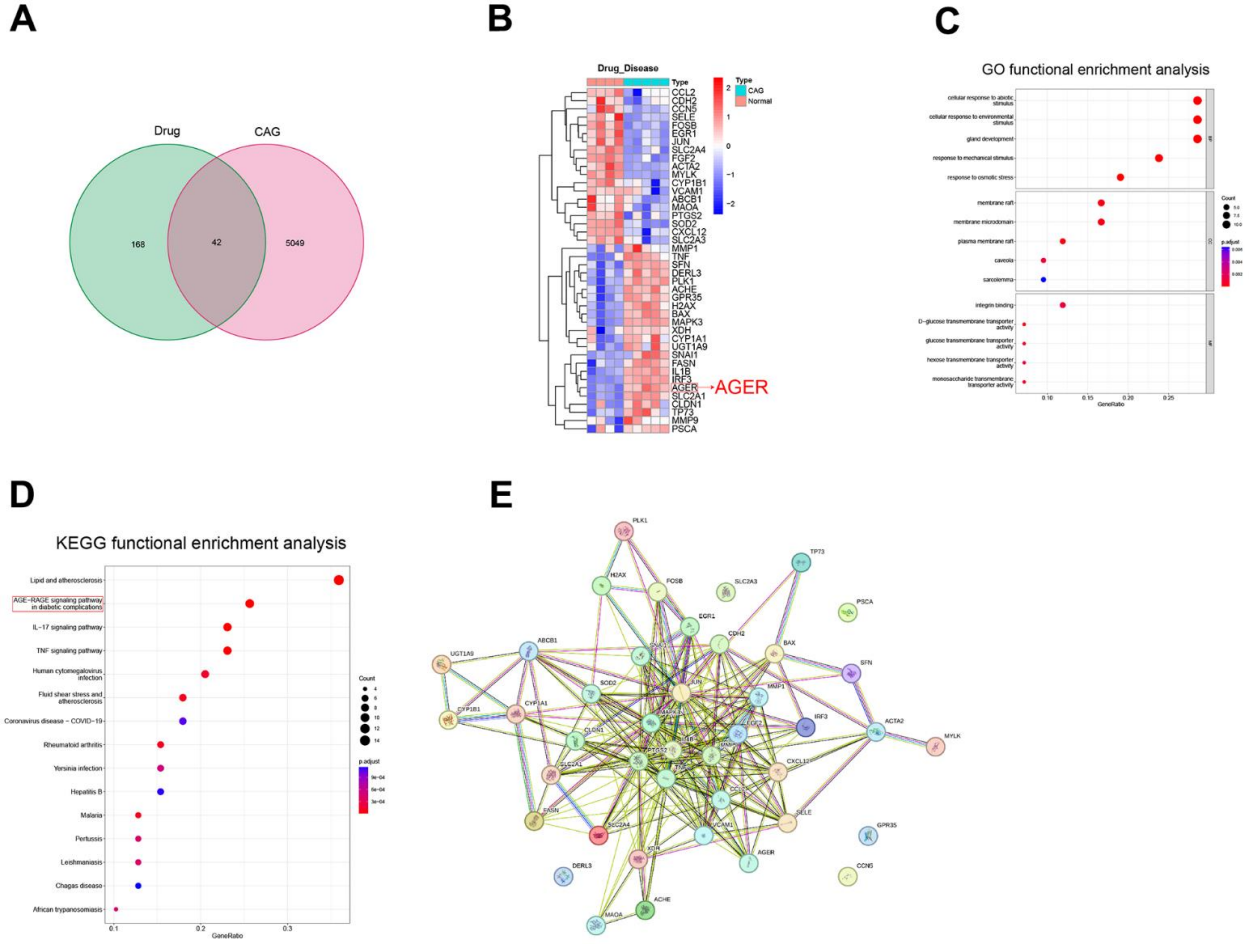
**Screening of potential therapeutic targets for luteolin.** (**A**) Drug targets and CAG differential genes were intersected to draw a Wayne diagram. (**B**) Heat map of intersecting gene clustering. (**C**) GO functional enrichment analysis. (**D**) KEGG functional enrichment analysis of intersecting genes. (**E**) Intersecting gene plot of the PPI network diagrams. CAG, chronic atrophic gastritis; GO, gene ontology; KEGG, Kyoto Encyclopedia of Genes and Genomes; PPI, protein-protein interaction.

**Figure 2:**
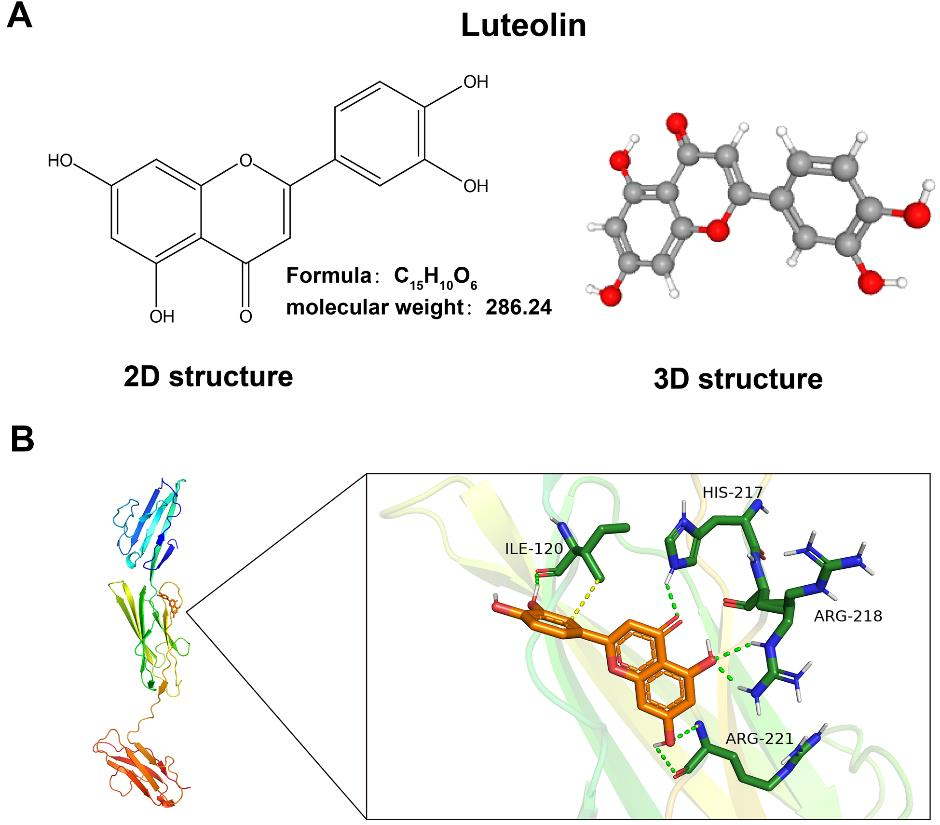
**Luteolin has a binding site with AGRE.** (**A**) 2D and 3D molecular structures and relative molecular mass of luteolin. (**B**) Schematic of luteolin docking with AGRE.

We downloaded the luteolin-predicted target information from the ETCM database to identify gene intersections with the above differential genes. By plotting a Wayne diagram (Figure 1A), we identified 42 intersecting genes. Next, we plotted the clustering heatmap of the intersecting genes (Figure 1B), GO functional enrichment analysis (Figure 1C), KEGG functional enrichment analysis (Figure 1D), and a protein-protein interaction (PPI) network map of the intersecting genes (Figure 1E). We found that the differential genes were mainly enriched in the AGE-RAGE signaling pathway, and RAGE proteins were aberrantly expressed in the CAG disease dataset. RAGE is a potential target of luteolin in the treatment of CAG. The 2D and 3D molecular formulae of luteolin are shown in Figure 2A. We simulated the molecular docking of luteolin with RAGE using a computer, and the results showed a binding pattern of AGER and luteolin as shown in Figure 2B, the binding scores reflect the binding strength between the receptor and ligand. The free energy of binding calculated from the docking of AGER and luteolin was -6.5 kcal/mol, in which the binding of luteolin to AGER on the ILE120, HIS217, ARG218, and ARG221 contributes to the tight binding of proteins and compounds. In addition, luteolin can form hydrophobic interactions with ILE120, which further indicates the strong binding ability between luteolin and RAGE (Figure 2B).

### Luteolin ameliorates gastric injury and inflammation in CAG rats

**Figure 3:**
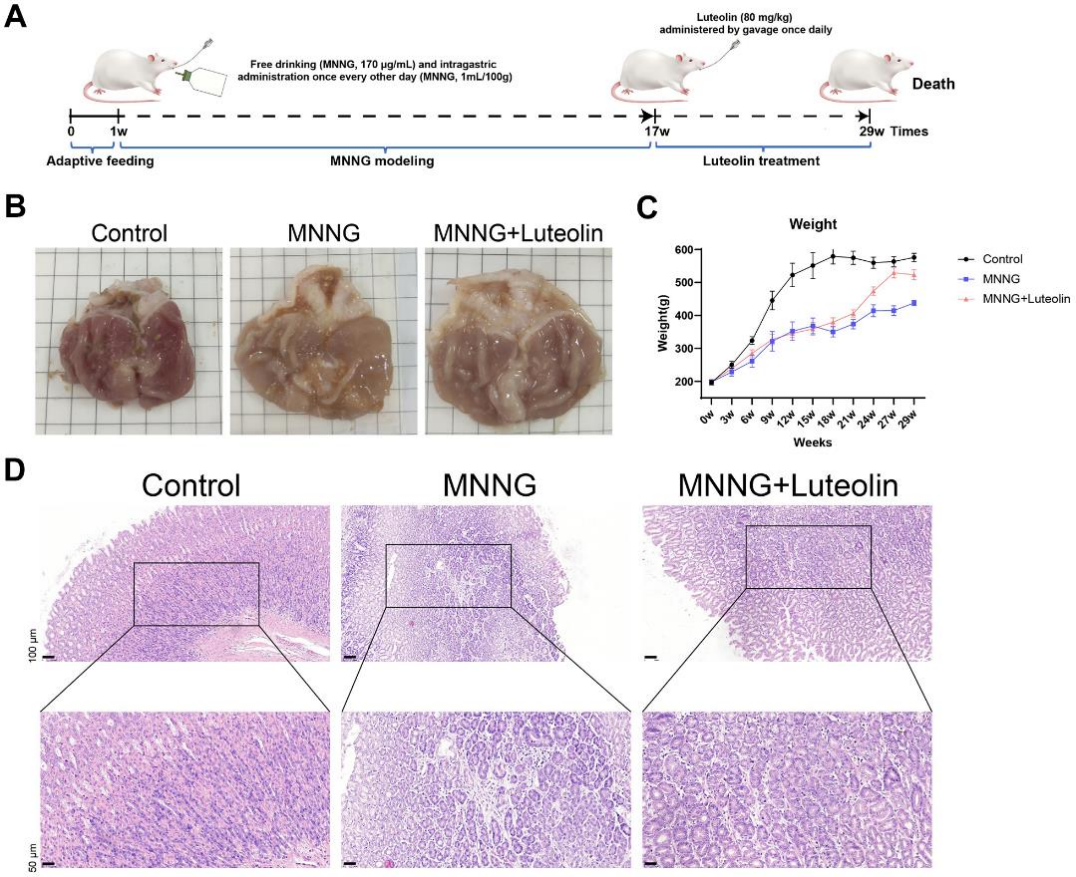
**Luteolin has a therapeutic effect on rat CAG *in vivo*.** (**A**) CAG animal modeling and treatment flowchart. (**B**) Top view of the rat gastric tissue anatomy. (**C**) Body weight curves of the rats. (**D**) HE staining of rat gastric tissues. CAG, chronic atrophic gastritis; HE, hematoxylin and eosin.

The MNNG model of CAG rats and subsequent luteolin treatment process are detailed in Figure 3A. The stomach tissue considerably improved after luteolin treatment (Figure 3B). We also observed the body weight changes of the rats during the modeling and treatment periods and found that the body weight of the rats in the MNNG-induced CAG model group increased slowly compared with that of the control group, whereas the body weight of the luteolin-treated group tended to increase faster than that of the model group, which proved that luteolin had a therapeutic effect, leading to a convergence of body weights in the luteolin-treated group compared with that of the normal rats (Figure 3C). Hematoxylin and eosin (HE) staining results clearly showed that the gastric mucosal intrinsic glands were significantly reduced in the CAG model group, with some tissue inflammation. Gastric mucosal glands were increased, and inflammation was reduced in the luteolin-treated group (Figure 3D).

**Figure 4:**
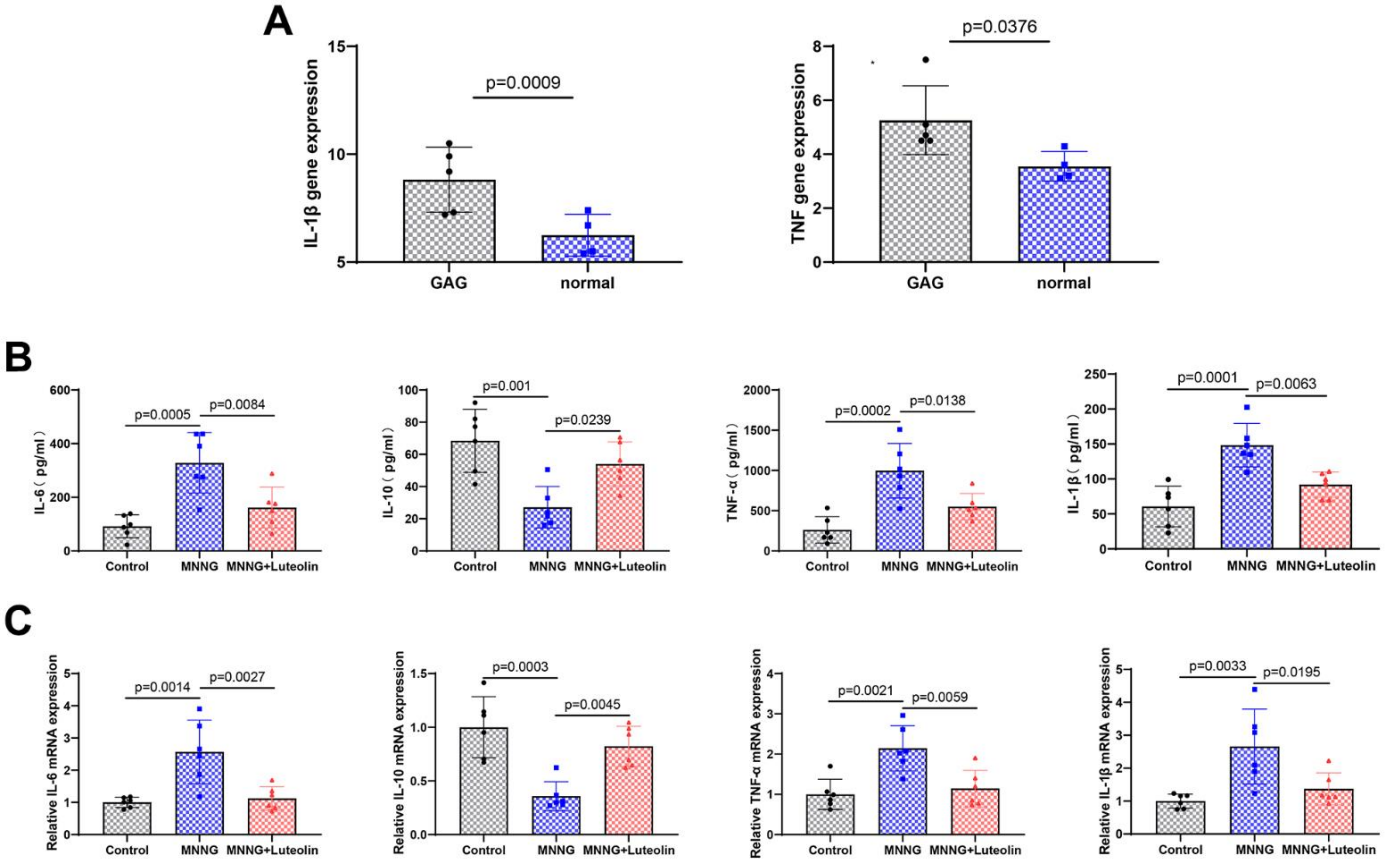
**Luteolin can reduce the inflammatory response in the gastric tissue of CAG rats.** (**A**) Expression of IL-1 β and TNF-α in the CAG dataset. (**B**) Changes in Il-6, Il-10, Tnf-α, and Il-1β levels in rat serum were determined by ELISA experiments. (**C**) The level of mRNA changes in *Il-6, Il-10, TNF-a*, and *Il-1b* after luteolin treatment was determined by qPCR.

We found that the expression levels of both IL-1β and TNF-α were elevated in CAG (Figure 4A). To further verify the anti-inflammatory effects of luteolin on CAG gastric tissues, we measured the expression of relevant inflammatory factors in rat serum using ELISA. The expression of IL-6, TNF-α, and IL-1β was significantly elevated and the expression level of IL-10 was reduced in the CAG group, whereas luteolin treatment was able to significantly attenuate the expression levels of IL-6, TNF-α, and IL-1β and enhance the expression level of IL-10 (Figure 4B). In addition, we also detected the changes in the mRNA expression of inflammatory factors by qPCR. Luteolin treatment significantly reduced the mRNA expression levels of *IL-6, TNF-α,* and *IL-1β*, and enhanced the expression level of *IL-10* mRNA (Figure 4C). These results further confirm the anti-inflammatory effects of luteolin.

### Luteolin can inhibit the ferroptosis in the gastric tissue of CAG rats

**Figure 5:**
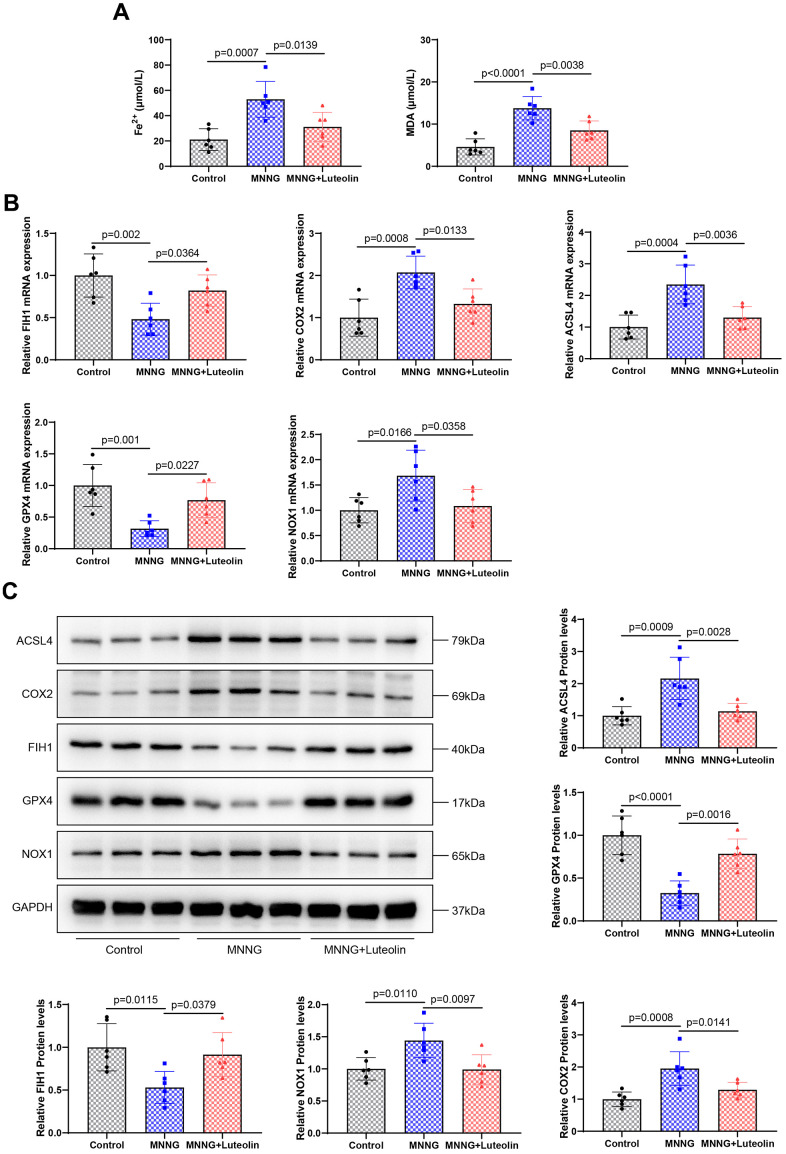
**Luteolin inhibits ferroptosis in rat gastric tissue.** (**A**) Changes in the levels of Fe^2+^ and MDA in gastric tissues of luteolin-treated rats. (**B**) Changes in the mRNA expression of FIH1, COX2, ACSL4, GPX4, and NOX1 were detected using qPCR. (**C**) Western blot analysis of the protein expression levels of FIH1, COX2, ACSL4, GPX4, and NOX1.

After we confirmed that luteolin was able to inhibit inflammation in the CAG model, to further elaborate on the pharmacological effects of luteolin, we verified the effects of luteolin treatment on ferroptosis in gastric tissues of the CAG model by observing ferroptosis-related indices. We found that the expression levels of MDA and Fe2+ were significantly increased in the CAG model group, whereas luteolin treatment downregulated the expression levels of MDA and Fe^2+^ (Figure 5A). Next, we detected the expression of ferroptosis-related indexes by qPCR (Figure 5B) and western blotting (Figure 5C). The results showed that the protein and mRNA expression levels of ACSL4, COX2, and NOX1 ferroptosis inducers were significantly increased in the CAG model group, while the protein and mRNA expression levels of the ferroptosis inhibitors, FIH1 and GPX4, were significantly decreased, suggesting that there was obvious ferroptosis in the gastric tissues of the CAG model group. Luteolin treatment significantly reduced ACSL4, COX2, and NOX1, and enhanced FIH1 and GPX4 protein and mRNA expression levels, which suggests that we luteolin can inhibit the ferroptosis of gastric tissues caused by the CAG model, and then reduce the gastric tissue damage.

### Luteolin inhibits the AGE-RAGE signaling pathway

**Figure 6:**
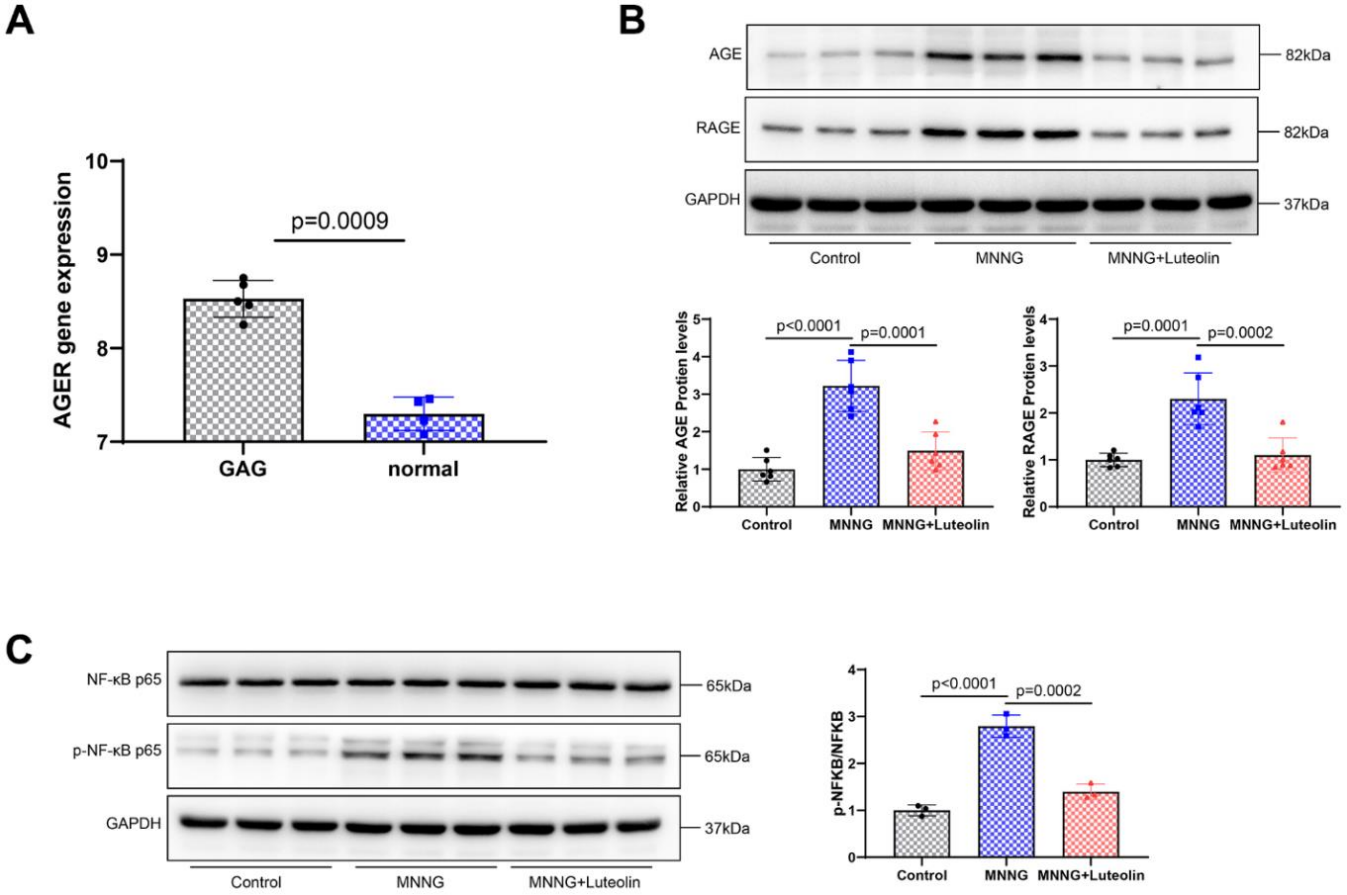
**Luteolin inhibits the activity of the AGE-RAGE signaling pathway.** (**A**) RAGE expression in the CAG disease database. (**B**) Western blot. The protein expression levels of AGE and RAGE were determined. (**C**) Western blot assay was used to detect the protein expression levels of NF-κB p65 and p-NF-κB p65.

We found that RAGE was abnormally expressed in CAG disease (Figure 6A) to further elucidate the mechanism underlying the pharmacological effects of luteolin, changes in pathway-related proteins were detected using western blotting. CAG was able to enhance the expression levels of AGE and RAGE proteins. The expression levels of AGE and RAGE proteins significantly decreased after luteolin treatment (Figure 6B). We also examined NF-κB p65 and p-NF-κB p65, related factors downstream of the AGE-RAGE signaling pathway. The result showed that CAG upregulated p-NF-κB p65/NF-κB p65, while luteolin was able to inhibit the expression level of p-NF-κB p65/NF-κB p65 (Figure 6C). Therefore, it was confirmed that luteolin inhibits the activity of the AGE-RAGE signaling pathway.

## DISCUSSION

To reduce the incidence of gastric cancer, it is crucial to prevent precancerous lesions in advance, and preventing chronic obscene gastritis is an important means to reduce the incidence of gastritis [21]. Recently, Chinese herbal medicines have made rapid progress in the treatment of CAG and are important in the development of CAG-specific drugs [22]. Luteolin has the natural advantages of low toxicity and high efficacy and has become a candidate drug for a variety of diseases [23]; its derivatives also show great potential in the field of diseases [24]. Luteolin has not previously been reported in CAG treatment. Based on bioinformatic analysis, we found that the differences between luteolin and CAG diseases were mainly concentrated in the AGE-RAGE signaling pathway, and luteolin also had strong binding sites for RAGE. RAGE is a potential therapeutic target for luteolin, which is consistent with predictions by others [13]. To our knowledge, the specific mechanism of the AGE-RAGE signaling pathway in CAG has not been reported, and AGE-RAGE signaling pathway is a malignant factor in various human diseases, including cancer, cardiovascular diseases, and nervous system diseases [25]. Abnormal RAGE expression was observed in the CAG disease database. Luteolin treatment significantly inhibited the activity of the AGE-RAGE signaling pathway and reduced the inflammatory response in the serum of CAG rats. Luteolin shows a strong anti-inflammatory effect, which has been confirmed in other diseases.

CAG diseases are usually accompanied by ferroptosis in the gastric tissue, and CAG-induced ferroptosis is characterized by decreased levels of GPX4 and FTH and increased levels of 4-HNE [17]. Ferrimodulin is mainly located in parietal cells and is elevated in CAG gastric tissue [18]. Wang et al. used the STRING database to construct an interaction network of potential target genes for age-related maculopathy, ferroptosis, and *Salvia miltiorrhiza*/*Fructus lycii*. Notably, luteolin has a strong enrichment relationship with genes related to ferroptosis [26]. In our study, luteolin significantly inhibited the expression of Fe^2+^ and MDA in CAG rat stomach tissues, upregulated FIH1 and GPX4, downregulated the expression of ACSL4, COX2, and NOX1 protein and mRNA, and inhibited ferroptosis in stomach tissues. Notably, luteolin-mediated ferroptosis has different effects on different cells. Luteolin can induce ferroptosis in cancer cells such as prostate cancer [27], colorectal cancer [28], and renal cell carcinoma [29], while inhibiting ferroptosis in normal cells [19, 30]. Ferroptosis is mainly caused by the action of bivalent iron ion or ester oxygenase, which catalyzes the highly expressed unsaturated fatty acids on the cell membrane and causes liposome peroxidation, thus inducing cell death [31]. We suspect that this may be different from the metabolic level of ferroptosis in cancer and normal cells, as well as the level of Fe^2+^ ions produced by cancer cells [32], resulting in different effects of luteolin on ferroptosis in different cells. This theory needs to be tested in future experiments.

In conclusion, we demonstrated for the first time that luteolin inhibited the expression of the AGE-RAGE signaling pathway, inhibited ferroptosis in gastric tissue, and alleviated gastric tissue damage and inflammation in CAG models. Although our results were encouraging, there are some limitations. The relationship between luteolin inhibition of ferroptosis and the AGE-RAGE signaling pathway needs to be further verified by reverse validation experiments. Additionally, the metabolic processes of luteolin in the body are unknown and may affect its efficacy and safety. The anti-inflammatory mechanism of luteolin in CAG is unclear; subsequent studies should compare the therapeutic effects of luteolin with those of anti-inflammatory drugs. Therefore, future studies should focus on the metabolic process of luteolin *in vivo* and elucidate the in-depth mechanism of luteolin treatment of CAG, inhibition of ferroptosis, and resistance to inflammation to provide more information for its clinical application.

In summary, the protective and therapeutic effects of luteolin on CAG via the inhibition of AGE-RAGE signaling pathways have been demonstrated. An in-depth study and exploration of the mechanism of action are expected to provide new strategies and methods for the prevention and treatment of CAG. However, more attention is needed regarding the bioavailability of luteolin and its metabolic processes *in vivo* to provide more information for its clinical application.

## MATERIALS AND METHODS

### GSE153224 and GSE191139 datasets screened for differential genes

The GSE153224 and GSE191139 database datasets were downloaded from the GEO database (https://www.ncbi.nlm.nih.gov/gds) and analyzed using the R packages “FactoMineR”, “factoextra”, and performed principal component analysis (PCA). Differential expression between the two groups was analyzed using the “limma” package. A total of 5091 differentially expressed genes were obtained by screening according to a p-value < 0.05 and |logFC| > 1. The R package “ggplot2” was used to map the differentially expressed genes. The R package “pheatmap” was used to map the top 20 differentially expressed genes. The 5091 genes analyzed in the GSE153224 and GSE191139 datasets were enriched with GO and KEGG functions using the R package “clusterProfiler”, and the top results were displayed.

### Luteolin screening of potential therapeutic targets

In the CTD database (https://ctdbase.org/), “Luteolin” was used as the key word and downloaded. The intersection of the Luteolin target with the differentially expressed genes obtained above resulted in 42 intersection genes. The R package “pheatmap” was used to map the top 20 differentially expressed genes. For the intersection genes obtained above, GO and KEGG functional enrichment analysis were performed using the R package “clusterProfiler”, and the top ranked results were presented.

### Luteolin docked with AGER molecules

First, the 3D structure of AGER (Protein Data Bank [Protein Data Bank, PDB] ID:4P2Y) was obtained from the PDB, and the SDF format of luteolin was obtained from the PubChem database (https://pubchem.ncbi.nlm.nih.gov) and converted to the PDB format using Open Babel. Proteins were dehydrogenated and charges were calculated and converted to pdbqt format using Autodocktools 1.5.7 software. The ligands were hydrogenated, the torsional forces were determined, and converted to the pdbqt format. Docking box coordinates were determined and molecular docking operations were performed using AutoDock Vina software. pymol 2.1.0 was used for visualization to obtain 3D analytical maps.

### *In vivo* experiment

Eighteen male Sprague-Dawley rats aged six to eight weeks, were kept at the Hebei University of Traditional Chinese Medicine. Adequate sterile feed and water were provided, and the formal experiment began after 7 days of feeding. The mice were randomly divided into three groups: control group (n = 6), 1-Methyl-3-nitro-1-nitrosoguanidine (MNNG) group (n = 6), and MNNG+Luteolin group (n = 6). According to the previous modeling method for chronic atrophic gastritis [33, 34], the control group had free access to drinking water and food, while the other groups had free access to MNNG (170 μg/mL) and irregular diet, and were given intragastric administration (MNNG, 170 μg/mL) every other day for 16 weeks. After modeling was completed, the MNNG+Luteolin group was intra-gastrically administered luteolin (80 mg/kg) once daily for 12 consecutive weeks [35, 36]. The weight changes in the rats were observed weekly. After treatment with luteolin, the rats in each group were euthanized by intraperitoneal injection of excessive anesthesia. The gastric tissues of the rats were subjected to pathological staining and molecular biology experiments. Luteolin (HY-N0162) and MNNG (HY-128612) were purchased from MedChem Express Biotechnology, Inc. (USA).

### qPCR

Fresh rat gastric tissue was extracted from each group using an appropriate amount of TRIzol (Solarbio Life Sciences, R1100, China) solution, and total tissue RNA was extracted according to the manufacturer’s instructions. RNA concentration was determined using a NanoDrop One (Thermo Fisher Scientific, 840-317400, China). cDNA was synthesized from reverse transcribed RNA using a first-strand synthesis kit (Solarbio Life Sciences, K16225). The one-step RT-qPCR RTase mixture (Solarbio Life Sciences, T2210) was added, and the fluorescence quantitative PCR instrument (Thermo Fisher Scientific, ABI 7900HT, 4351405) was set under the following conditions: 50° C for 20 min, 95° C for 3 min, followed by: 45 cycles of 95° C for 20 s, 60° C for 30 s. Machine data were saved and analyzed using the software provided by the ABI 7900HT fluorescence quantitative PCR instrument. The sequences of the QPCR primers are detailed in Table 1.

**Table 1:** Primer sequences of the qPCR constructs.

**Name of gene**	**Sequence (5' to 3')**
Forward ACSL4	CTGGGATCCAAGCCAGAAAA
Reverse ACSL4	GCATCATCACTCCCTTGGGG
Forward COX2	AGGAGCATCCTGAGTGGGAT
Reverse COX2	AGGAGCATCCTGAGTGGGAT
Forward FIH1	TCCTCCGGATCAGTTCGAGT
Reverse FIH1	AGTTGGGGAAGCGCTCATAG
Forward GPX4	GCCGGCTACAATGTCAGGTT
Reverse GPX4	CATGGGACCATAGCGCTTCA
Forward NOX1	GTTTCTTGGTTGGGGCTGAACA
Reverse NOX1	TTCGACACACAGGAATCAGGA
Forward IL-6	CACTTCACAAGTCGGAGGCT
Reverse IL-6	TCTGACAGTGCATCATCGCT
Forward IL-10	CCAGCAAAGGCCATTCCATC
Reverse IL-10	TGGCAACCCAAGTAACCCTT
Forward IL-1β	GTGCTGTCTGACCCATGTGA
Reverse IL-1β	GATTCTTCCCCTTGAGGCCC
Forward TNF-α	CGTCAGCCGATTTGCCATTT
Reverse TNF-α	TCCCTCAGGGGTGTCCTTAG
Forward GAPDH	CATGGCCTTCCGTGTTCCTA
Reverse GAPDH	ACAGGAGACAACCTGGTCCT

### Western blot assay

Fresh rat stomach tissue from each group was extracted and incubated with tissue lysates at room temperature for 30 min to extract the protein solution. The protein concentration was determined using a BCA kit (Solarbio Life Sciences, PC0020). The voltage was set to 120 V for gel electrophoresis and the gel was transferred to a PVDF membrane after 75 min. Subsequently, the voltage was adjusted to 200 V. After 30 min, the PVDF membranes were incubated with 5% skim milk powder for 2 h. After incubation, the membrane was washed thrice with TBST, and the primary antibody was washed thrice with TBST after overnight incubation. After incubation with the secondary antibody for 2 h, a chemiluminescent solution was added and incubated for 10 s. The membranes were imaged using a chemiluminescence instrument (Thermo Fisher Scientific) to preserve the images for subsequent statistical analyses. NF-κB p65 (#AF5006, 1:1000), p-NF-κB p65 (#AF2006, 1:1000), ACSL4 (DF12141, 1:1000), COX2 (AF7003, 1:1000), FIH1 (DF7354, 1:1000), GPX4 (DF67012, 1:1000), NOX1 (DF8684, 1:1000), GAPDH (#AF7021, 1:5000), AGRE (BF8005, 1:1000), AGE (AF2006, 1:1000), Goat Anti-Rabbit IgG (H+L) HRP (#S0001, 1:10000), and goat anti-mouse IgG (H+L) HRP (#S0002, 1:10000) were purchased from Affinity Biosciences Company, Jiangsu, China. AGE (ab23722) and RAGE (ab216329) were purchased from Abcam, UK.

### HE stain

Rat stomach tissues of appropriate size were fixed in a tissue fixative and dehydrated with an alcohol gradient for transparency. The tissues were then embedded in dipping wax, cooled, sectioned, stained according to the procedure of the HE staining kit (Solarbio Life Sciences, G1120), covered with coverslips, sealed, dried naturally, photographed with a microscope (Olympus Corporation, BX53), and preserved for subsequent analyses.

### Fe^2+^ and MDA content assay

Fresh stomach tissues were extracted from each group. Cell samples were processed according to the requirements of the Iron Content Assay Kit (Merck, MAK025, Germany), the concentration of Fe2+ in the cells was detected, and the OD value (593 nm) was detected using SpectraMax Mini multifunctional enzyme marker. The cells were processed according to the requirements of the MDA assay kit (Beyotime Biotechnology, S0131S, China), and OD value (532 nm) was detected using a SpectraMax Mini multifunctional enzyme marker.

### Statistical analysis

GraphPad Prism 9.5.0 software was adopted for data analysis. The unpaired tissues were assessed by the Wilcoxon rank-sum test, and other data were evaluated using one-way ANOVA, followed by the post hoc comparisons with Tukey’s honestly significant difference test. *P*<0.05 indicates the difference is statistically significant.

### Availability of data and materials

The datasets used and/or analyzed during the current study are available from the corresponding author on reasonable request.

## Supplementary Material

Supplementary Figure 1

## References

[1] Yin Y, Liang H, Wei N, Zheng Z. Prevalence of chronic atrophic gastritis worldwide from 2010 to 2020: an updated systematic review and meta-analysis. Ann Palliat Med. 2022; 11:3697–703. 10.21037/apm-21-1464 36635994

[2] Osmola M, Hemont C, Chapelle N, Vibet MA, Tougeron D, Moussata D, Lamarque D, Bigot-Corbel E, Masson D, Blin J, Leroy M, Josien R, Mosnier JF, et al. Atrophic Gastritis and Autoimmunity: Results from a Prospective, Multicenter Study. Diagnostics (Basel). 2023; 13:1599. 10.3390/diagnostics13091599 37174990 PMC10178247

[3] Shah SC, Piazuelo MB, Kuipers EJ, Li D. AGA Clinical Practice Update on the Diagnosis and Management of Atrophic Gastritis: Expert Review. Gastroenterology. 2021; 161:1325–32.e7. 10.1053/j.gastro.2021.06.078 34454714 PMC8740554

[4] Neumann WL, Coss E, Rugge M, Genta RM. Autoimmune atrophic gastritis--pathogenesis, pathology and management. Nat Rev Gastroenterol Hepatol. 2013; 10:529–41. 10.1038/nrgastro.2013.101 23774773

[5] Wang L, Ding X, Li P, Zhang F, Ru S, Wang F, Li L. Efficacy and safety of Weifuchun tablet for chronic atrophic gastritis: A systematic review and meta-analysis. PLoS One. 2023; 18:e0284411. 10.1371/journal.pone.0284411 37053262 PMC10101393

[6] Rossi RE, Elvevi A, Sciola V, Mandarino FV, Danese S, Invernizzi P, Massironi S. Paradoxical association between dyspepsia and autoimmune chronic atrophic gastritis: Insights into mechanisms, pathophysiology, and treatment options. World J Gastroenterol. 2023; 29:3733–47. 10.3748/wjg.v29.i23.3733 37398891 PMC10311608

[7] Dottori L, Corleone Tsar’kov D, Dilaghi E, Pivetta G, Scalamonti S, Ligato I, Esposito G, Annibale B, Lahner E. Efficacy and Safety of Intravenous Ferric Carboxymaltose Treatment of Iron Deficiency Anaemia in Patients with Corpus Atrophic Gastritis: A Retrospective Study. Nutrients. 2023; 15:4199. 10.3390/nu15194199 37836482 PMC10574262

[8] Issever K, Kuloglu E, Muhtaroglu A, Seker D, Kotur O, Dulger AC. The Effect of Direct Oral Anticoagulants on Gastric Mucosa and Helicobacter Pylori Prevalence in Dyspeptic Patients: A Retrospective Cross-Sectional Study. Cureus. 2023; 15:e46477. 10.7759/cureus.46477 37927617 PMC10623502

[9] Yazarlu O, Iranshahi M, Kashani HR, Reshadat S, Habtemariam S, Iranshahy M, Hasanpour M. Perspective on the application of medicinal plants and natural products in wound healing: A mechanistic review. Pharmacol Res. 2021; 174:105841. 10.1016/j.phrs.2021.105841 34419563

[10] He YQ, Zhou CC, Yu LY, Wang L, Deng JL, Tao YL, Zhang F, Chen WS. Natural product derived phytochemicals in managing acute lung injury by multiple mechanisms. Pharmacol Res. 2021; 163:105224. 10.1016/j.phrs.2020.105224 33007416 PMC7522693

[11] Prasher P, Sharma M, Singh SK, Gulati M, Chellappan DK, Zacconi F, De Rubis G, Gupta G, Sharifi-Rad J, Cho WC, Dua K. Luteolin: a flavonoid with a multifaceted anticancer potential. Cancer Cell Int. 2022; 22:386. 10.1186/s12935-022-02808-3 36482329 PMC9730645

[12] Gendrisch F, Esser PR, Schempp CM, Wölfle U. Luteolin as a modulator of skin aging and inflammation. Biofactors. 2021; 47:170–80. 10.1002/biof.1699 33368702

[13] Duan ZL, Wang YJ, Lu ZH, Tian L, Xia ZQ, Wang KL, Chen T, Wang R, Feng ZY, Shi GP, Xu XT, Bu F, Ding Y, et al. Wumei Wan attenuates angiogenesis and inflammation by modulating RAGE signaling pathway in IBD: Network pharmacology analysis and experimental evidence. Phytomedicine. 2023; 111:154658. 10.1016/j.phymed.2023.154658 36706698

[14] Weng J, Wu XF, Shao P, Liu XP, Wang CX. Medicine for chronic atrophic gastritis: a systematic review, meta- and network pharmacology analysis. Ann Med. 2023; 55:2299352. 10.1080/07853890.2023.2299352 38170849 PMC10769149

[15] Shi M, Chen Z, Gong H, Peng Z, Sun Q, Luo K, Wu B, Wen C, Lin W. Luteolin, a flavone ingredient: Anticancer mechanisms, combined medication strategy, pharmacokinetics, clinical trials, and pharmaceutical researches. Phytother Res. 2024; 38:880–911. 10.1002/ptr.8066 38088265

[16] Pope LE, Dixon SJ. Regulation of ferroptosis by lipid metabolism. Trends Cell Biol. 2023; 33:1077–87. 10.1016/j.tcb.2023.05.003 37407304 PMC10733748

[17] Zhao Y, Zhao J, Ma H, Han Y, Xu W, Wang J, Cai Y, Jia X, Jia Q, Yang Q. High Hepcidin Levels Promote Abnormal Iron Metabolism and Ferroptosis in Chronic Atrophic Gastritis. Biomedicines. 2023; 11:2338. 10.3390/biomedicines11092338 37760781 PMC10525531

[18] Guo Y, Jia X, Du P, Wang J, Du Y, Li B, Xue Y, Jiang J, Cai Y, Yang Q. Mechanistic insights into the ameliorative effects of Xianglianhuazhuo formula on chronic atrophic gastritis through ferroptosis mediated by YY1/miR-320a/TFRC signal pathway. J Ethnopharmacol. 2024; 323:117608. 10.1016/j.jep.2023.117608 38158098

[19] Gao S, Gao Y, Cai L, Qin R. Luteolin attenuates Staphylococcus aureus-induced endometritis through inhibiting ferroptosis and inflammation via activating the Nrf2/GPX4 signaling pathway. Microbiol Spectr. 2024; 12:e0327923. 10.1128/spectrum.03279-23 38169293 PMC10846197

[20] Wang IC, Lin JH, Lee WS, Liu CH, Lin TY, Yang KT. Baicalein and luteolin inhibit ischemia/reperfusion-induced ferroptosis in rat cardiomyocytes. Int J Cardiol. 2023; 375:74–86. 10.1016/j.ijcard.2022.12.018 36513286

[21] Pimentel-Nunes P, Libânio D, Marcos-Pinto R, Areia M, Leja M, Esposito G, Garrido M, Kikuste I, Megraud F, Matysiak-Budnik T, Annibale B, Dumonceau JM, Barros R, et al. Management of epithelial precancerous conditions and lesions in the stomach (MAPS II): European Society of Gastrointestinal Endoscopy (ESGE), European Helicobacter and Microbiota Study Group (EHMSG), European Society of Pathology (ESP), and Sociedade Portuguesa de Endoscopia Digestiva (SPED) guideline update 2019. Endoscopy. 2019; 51:365–88. 10.1055/a-0859-1883 30841008

[22] Yang L, Liu X, Zhu J, Zhang X, Li Y, Chen J, Liu H. Progress in traditional Chinese medicine against chronic gastritis: From chronic non-atrophic gastritis to gastric precancerous lesions. Heliyon. 2023; 9:e16764. 10.1016/j.heliyon.2023.e16764 37313135 PMC10258419

[23] Huang L, Kim MY, Cho JY. Immunopharmacological Activities of Luteolin in Chronic Diseases. Int J Mol Sci. 2023; 24:2136. 10.3390/ijms24032136 36768462 PMC9917216

[24] Manzoor MF, Ahmad N, Ahmed Z, Siddique R, Zeng XA, Rahaman A, Muhammad Aadil R, Wahab A. Novel extraction techniques and pharmaceutical activities of luteolin and its derivatives. J Food Biochem. 2019; 43:e12974. 10.1111/jfbc.12974 31489656

[25] Bhattacharya R, Alam MR, Kamal MA, Seo KJ, Singh LR. AGE-RAGE axis culminates into multiple pathogenic processes: a central road to neurodegeneration. Front Mol Neurosci. 2023; 16:1155175. 10.3389/fnmol.2023.1155175 37266370 PMC10230046

[26] Wang L, Zhang C, Pang L, Wang Y. Integrated network pharmacology and experimental validation to explore the potential pharmacological mechanism of Qihuang Granule and its main ingredients in regulating ferroptosis in AMD. BMC Complement Med Ther. 2023; 23:420. 10.1186/s12906-023-04205-3 37990310 PMC10664676

[27] Fu W, Xu L, Chen Y, Zhang Z, Chen S, Li Q, You X. Luteolin induces ferroptosis in prostate cancer cells by promoting TFEB nuclear translocation and increasing ferritinophagy. Prostate. 2024; 84:223–36. 10.1002/pros.24642 37904332

[28] Zheng Y, Li L, Chen H, Zheng Y, Tan X, Zhang G, Jiang R, Yu H, Lin S, Wei Y, Wang Y, Zhang R, Liu Z, Wu J. Luteolin exhibits synergistic therapeutic efficacy with erastin to induce ferroptosis in colon cancer cells through the HIC1-mediated inhibition of GPX4 expression. Free Radic Biol Med. 2023; 208:530–44. 10.1016/j.freeradbiomed.2023.09.014 37717793

[29] Han S, Lin F, Qi Y, Liu C, Zhou L, Xia Y, Chen K, Xing J, Liu Z, Yu W, Zhang Y, Zhou X, Rao T, Cheng F. HO-1 Contributes to Luteolin-Triggered Ferroptosis in Clear Cell Renal Cell Carcinoma via Increasing the Labile Iron Pool and Promoting Lipid Peroxidation. Oxid Med Cell Longev. 2022; 2022:3846217. 10.1155/2022/3846217 35656025 PMC9153929

[30] Li X, Qi H, Zhang X, Liang H, Zeng N. Jing-Fang n-butanol extract and its isolated JFNE-C inhibit ferroptosis and inflammation in LPS induced RAW264.7 macrophages via STAT3/p53/SLC7A11 signaling pathway. J Ethnopharmacol. 2023; 316:116689. 10.1016/j.jep.2023.116689 37315642

[31] Chen X, Yu C, Kang R, Kroemer G, Tang D. Cellular degradation systems in ferroptosis. Cell Death Differ. 2021; 28:1135–48. 10.1038/s41418-020-00728-1 33462411 PMC8027807

[32] Chen X, Kang R, Kroemer G, Tang D. Organelle-specific regulation of ferroptosis. Cell Death Differ. 2021; 28:2843–56. 10.1038/s41418-021-00859-z 34465893 PMC8481335

[33] Tong Y, Liu L, Wang R, Yang T, Wen J, Wei S, Jing M, Zou W, Zhao Y. Berberine Attenuates Chronic Atrophic Gastritis Induced by MNNG and Its Potential Mechanism. Front Pharmacol. 2021; 12:644638. 10.3389/fphar.2021.644638 33841162 PMC8026873

[34] Zhang J, Wang H. Morroniside protects against chronic atrophic gastritis in rat via inhibiting inflammation and apoptosis. Am J Transl Res. 2019; 11:6016–23. 31632569 PMC6789209

[35] Sun GB, Sun X, Wang M, Ye JX, Si JY, Xu HB, Meng XB, Qin M, Sun J, Wang HW, Sun XB. Oxidative stress suppression by luteolin-induced heme oxygenase-1 expression. Toxicol Appl Pharmacol. 2012; 265:229–40. 10.1016/j.taap.2012.10.002 23051850

[36] Tan X, Liu B, Lu J, Li S, Baiyun R, Lv Y, Lu Q, Zhang Z. Dietary luteolin protects against HgCl_2_-induced renal injury via activation of Nrf2-mediated signaling in rat. J Inorg Biochem. 2018; 179:24–31. 10.1016/j.jinorgbio.2017.11.010 29156292

