# Supplementary material for: Luteolin targets the AGE-RAGE signaling to mitigate inflammation and ferroptosis in chronic atrophic gastritis
Source: Aging (Albany NY). 2024 Jun 24;16(13):10918–30. doi: 10.18632/aging.205969 (PMC11272119; doi:10.18632/aging.205969)
Supplement: Supplementary Figure 1 [file aging-16-205969-s001.pdf]

SUPPLEMENTARY FIGURE

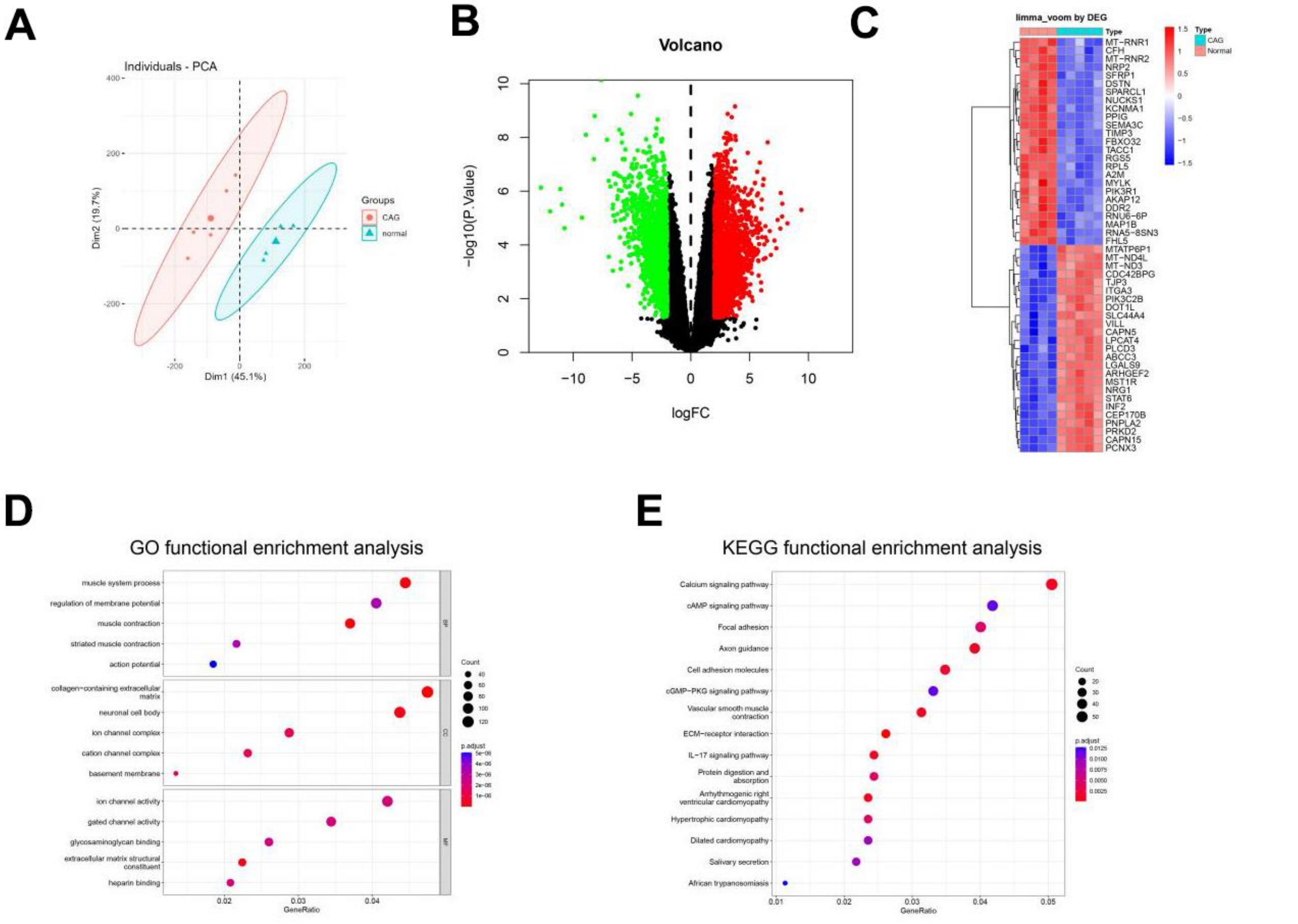

Supplementary Figure 1. CAG screen for differentially expressed genes. (**A**) GSE153224 and GSE191139 datasets were subjected to principal component analysis (PCA) for both sets of samples. (**B**) Differential genes were plotted in volcano plots. (**C**) Differential genes were plotted in clustered heat maps. (**D**) Differential gene GO functional enrichment analysis. (**E**) Differential gene KEGG functional enrichment analysis.
